# Neonatal Encephalopathic Cerebral Injury in South India Assessed by Perinatal Magnetic Resonance Biomarkers and Early Childhood Neurodevelopmental Outcome

**DOI:** 10.1371/journal.pone.0087874

**Published:** 2014-02-05

**Authors:** Peter J. Lally, David L. Price, Shreela S. Pauliah, Alan Bainbridge, Justin Kurien, Neeraja Sivasamy, Frances M. Cowan, Guhan Balraj, Manjula Ayer, Kariyapilly Satheesan, Sreejith Ceebi, Angie Wade, Ravi Swamy, Shaji Padinjattel, Betty Hutchon, Madhava Vijayakumar, Mohandas Nair, Krishnakumar Padinharath, Hui Zhang, Ernest B. Cady, Seetha Shankaran, Sudhin Thayyil

**Affiliations:** 1 Perinatal Neurology and Neonatology, Imperial College London, London, United Kingdom; 2 Medical Physics and Bioengineering, University College London Hospitals, London, United Kingdom; 3 Neonatal Medicine, Government Medical College, Kozhikode, Kerala, India; 4 Imperial College London, London, United Kingdom; 5 Neonatal Medicine, Manipal Hospital, Bangalore, Karnataka, India; 6 Imaging, Dr Shaj’s MRI and Research Centre, Kozhikode, Kerala, India; 7 Centre for Medical Image Computing, University College London, London, United Kingdom; 8 Neonatal-Perinatal Division, Wayne State University, Detroit, Massachusetts, United States of America; Vanderbilt University, United States of America

## Abstract

Although brain injury after neonatal encephalopathy has been characterised well in high-income countries, little is known about such injury in low- and middle-income countries. Such injury accounts for an estimated 1 million neonatal deaths per year. We used magnetic resonance (MR) biomarkers to characterise perinatal brain injury, and examined early childhood outcomes in South India.

**Methods:**

We recruited consecutive term or near term infants with evidence of perinatal asphyxia and a Thompson encephalopathy score ≥6 within 6 h of birth, over 6 months. We performed conventional MR imaging, diffusion tensor MR imaging and thalamic proton MR spectroscopy within 3 weeks of birth. We computed group-wise differences in white matter fractional anisotropy (FA) using tract based spatial statistics. We allocated Sarnat encephalopathy stage aged 3 days, and evaluated neurodevelopmental outcomes aged 3½ years using Bayley III.

**Results:**

Of the 54 neonates recruited, Sarnat staging was mild in 30 (56%); moderate in 15 (28%) and severe in 6 (11%), with no encephalopathy in 3 (6%). Six infants died. Of the 48 survivors, 44 had images available for analysis. In these infants, imaging indicated perinatal rather than established antenatal origins to injury. Abnormalities were frequently observed in white matter (n = 40, 91%) and cortex (n = 31, 70%) while only 12 (27%) had abnormal basal ganglia/thalami. Reduced white matter FA was associated with Sarnat stage, deep grey nuclear injury, and MR spectroscopy N-acetylaspartate/choline, but not early Thompson scores. Outcome data were obtained in 44 infants (81%) with 38 (79%) survivors examined aged 3½ years; of these, 16 (42%) had adverse neurodevelopmental outcomes.

**Conclusions:**

No infants had evidence for established brain lesions, suggesting potentially treatable perinatal origins. White matter injury was more common than deep brain nuclei injury. Our results support the need for rigorous evaluation of the efficacy of rescue hypothermic neuroprotection in low- and middle-income countries.

## Introduction

In high-income countries, neonatal encephalopathy occurs in 1 to 3 per 1000 live births; approximately 20 to 25% of the affected infants die, and 40% of the survivors have significant brain injury and lifelong disability [1], [2]. Magnetic resonance (MR) studies in the 1990s suggested that the brain injury in these infants is of perinatal origin, rather than being established antenatally, and is therefore potentially treatable [3]–[5]. This suggestion was followed by a number of randomised controlled trials in high-income countries, demonstrating a reduction in brain injury, and an improvement in survival with normal neurological outcomes following brain cooling therapy [6]–[9]. Cooling is now offered as standard of care therapy for neonatal encephalopathy of apparent hypoxic-ischaemic origin in high-income countries. Cerebral MR biomarkers, especially elevated proton spectroscopy lactate/N-acetylaspartate peak-area ratios in deep brain nuclei, and reduced white matter water-diffusion fractional anisotropy (FA) correlate well with adverse neurodevelopmental outcomes in these infants [10]–[12].

Approximately 99% of deaths (1 million deaths per year) from neonatal encephalopathy occur in low- and middle-income countries [13]. Thus, the global health benefit of cooling therapy is potentially far higher in low- and middle-income countries than in high-income. However, there are limited data on the pattern of brain injury and early childhood outcomes after neonatal encephalopathy in low- and middle-income countries. Before cooling therapy is evaluated as a potential treatment in low- and middle-income countries, it is crucial to understand the origins and nature of brain injury and early childhood neurodevelopmental outcomes in these populations.

In this study, we aimed to apply cerebral MR biomarkers to characterise the nature of brain injury in a cohort of term neonates with neonatal encephalopathy in South India.

## Methods

### Ethics Statement

The Government Medical College, Kozhikode Institutional Review Board and University College London Ethics Committee approved the study.

### Study Design

We prospectively screened for eligibility consecutive newborn infants admitted to Government Medical College, Kozhikode, Kerala, India, between July 2009 and December 2009. Infants requiring resuscitation at birth and/or Apgar score ≤5 at 5 minutes after birth and a Thompson encephalopathy score >5 [14] within 6 hours after birth, as assessed by a research physician, were recruited after parental consent. We excluded infants of <36 gestational weeks and/or of birth weight <1800 g, and those in whom a major congenital malformation was evident. Towards the second half of the study, some infants received whole body cooling using phase-changing material [15].

We performed infection screening (blood counts, blood culture, and plasma C–reactive protein) on all infants at admission and at age 3 days, and intravenous antibiotics were given if clinically indicated. For the purpose of the study sepsis was defined as clinical signs of infection as assessed by the attending clinician along with C-reactive protein >10 mg/l with or without positive blood culture, and treatment with antibiotics. We also measured renal and liver function, electrolytes, and coagulation profile soon after birth and again between 3 and 4 days of age, and blood glucose levels every 6 hours. An experienced neonatologist (MA) performed daily Thompson encephalopathy scoring until age 4 days, Sarnat staging [16] at age 3 days and a neurological examination at discharge [17]; infants were classified as abnormal if displaying alterations in alertness or tone, or neurological deficits on the discharge examination.

MR imaging examinations were performed using a 1.5 Tesla scanner (Siemens Avanto, Erlangen, Germany) between the ages of 1 to 3 weeks. The scanning protocol included 3D longitudinal relaxation time (T_1_)-weighted fast low-angle shot (FLASH), transverse relaxation time (T_2_)-weighted 2D fast spin-echo images (axial and coronal planes), diffusion tensor imaging (DTI; spin-echo echo-planar imaging sequence, 20 directions, b 0 & 1000 s/mm^2^, repetition time (TR)/echo time (TE) 2800 ms/94 ms, acquisition matrix 128×128, contiguous 5 mm thick axial slices) and proton MR spectroscopy (water-suppressed point-resolved spectroscopy (PRESS), TR/TE = 2290 ms/288 ms, 37×8 summed echo sub-spectra) in a single cubic voxel of 15×15×15 mm^3^ positioned in the left thalamus. Total MR imaging and spectroscopy scan duration was 35 minutes, during which heart rate and oxygen saturation were continuously monitored. Ear plugs were used for hearing protection throughout, and intra-nasal midazolam was administered if sedation was required.

### MR Data Analysis

An experienced perinatal neurologist (FMC) assessed the MR images according to a validated encephalopathy scoring system, masked to the clinical details, early childhood outcomes and other MR biomarker data but aware of the gestational age at birth and postnatal age at scan [18]. The nature of the brain injury was further classified to be either antenatal (e.g. cystic lesions, regions of tissue loss, longstanding haemorrhage, enlargement of ventricular and extra-cerebral spaces) or perinatal (e.g. acute evolving lesions, loss of cortical grey matter and white matter differentiation, brain swelling) [5]. Evidence for abnormal brain development was also assessed.

We analysed whole-brain white matter FA with tract-based spatial statistics (TBSS) using the Functional Magnetic Resonance Imaging of the Brain Software Library (FSL, Version 4.1) [19] and the Diffusion Tensor Imaging ToolKit (DTI-TK, Version 2.3.1) [20]. First, for each subject, diffusion-weighted data were corrected for motion and eddy current distortion, segmented to exclude extracerebral tissue, and used to reconstruct the diffusion tensor volume, with FSL. Second, the diffusion tensor volumes from all the subjects were spatially normalised with an optimized pipeline in DTI-TK. The pipeline uses the state-of-the-art diffusion tensor image registration algorithm [18] to provide the optimal alignment of white matter anatomy [21]. Finally, the spatially-normalised FA volumes were used to perform the TBSS analysis as previously described [22]. A lower FA threshold (>0.10) was required to identify the most prominent white matter tracts. Significance for each test was considered to be the presence of group-wise FA differences with p<0.05, corrected for multiple comparisons using Threshold-Free Cluster Enhancement [23].

We analysed MR spectra using the jMRUI spectroscopy package [24], and sub-spectra with motion artefacts were corrected or discarded prior to summation. Relative peak-areas of choline, creatine, lactate and N-acetylaspartate were determined using the AMARES spectrum fitting algorithm [25].

### Early Childhood Outcome Evaluation

An experienced UK and international trainer BH with the assistance of RS trained the local occupational therapist (JK) and a clinician (NS) to administer the Bayley III examination [26]. The parents were contacted by telephone or sent letters, asking them to attend the outpatient assessments unit at Government Medical College, Kozhikode between December 2012 and March 2013.

We performed a detailed neurological examination in all infants to identify cerebral palsy or other neurological deficits, and administered the Bayley III examination. In addition, we assessed the functional severity of cerebral palsy using the Gross Motor Function Classification System (GMFCS) [27], measured head circumference, height and weight, and assessed hearing and vision. The assessors were masked to the neonatal MR biomarker findings and neonatal clinical information, but were aware that the infants had met the initial recruitment criteria.

Abnormal outcome was classified as cerebral palsy of any severity, any form of visual (not correctable by glasses) or hearing impairment; evidence of seizures after the neonatal period and/or continued use of anti-epileptic medication at age 3½ years; slowed head growth (reduction of >2 standard deviations from head circumference centile at birth assessed using WHO 2006 head circumference charts, corrected for sex); or a composite motor score <82 or composite cognitive score <85 on Bayley III [28], allowing for differences in scores between the Bayley II and Bayley III examinations.

We used exact methods to calculate confidence intervals, and Chi-square tests and t-tests to examine statistical significance. The data were analysed using SPSS (Version 21, International Business Machines Corp, Armonk, New York) and MedCalc (Version 12.7.0, MedCalc Software, Ostend, Belgium).

## Results

**Figure 1:**
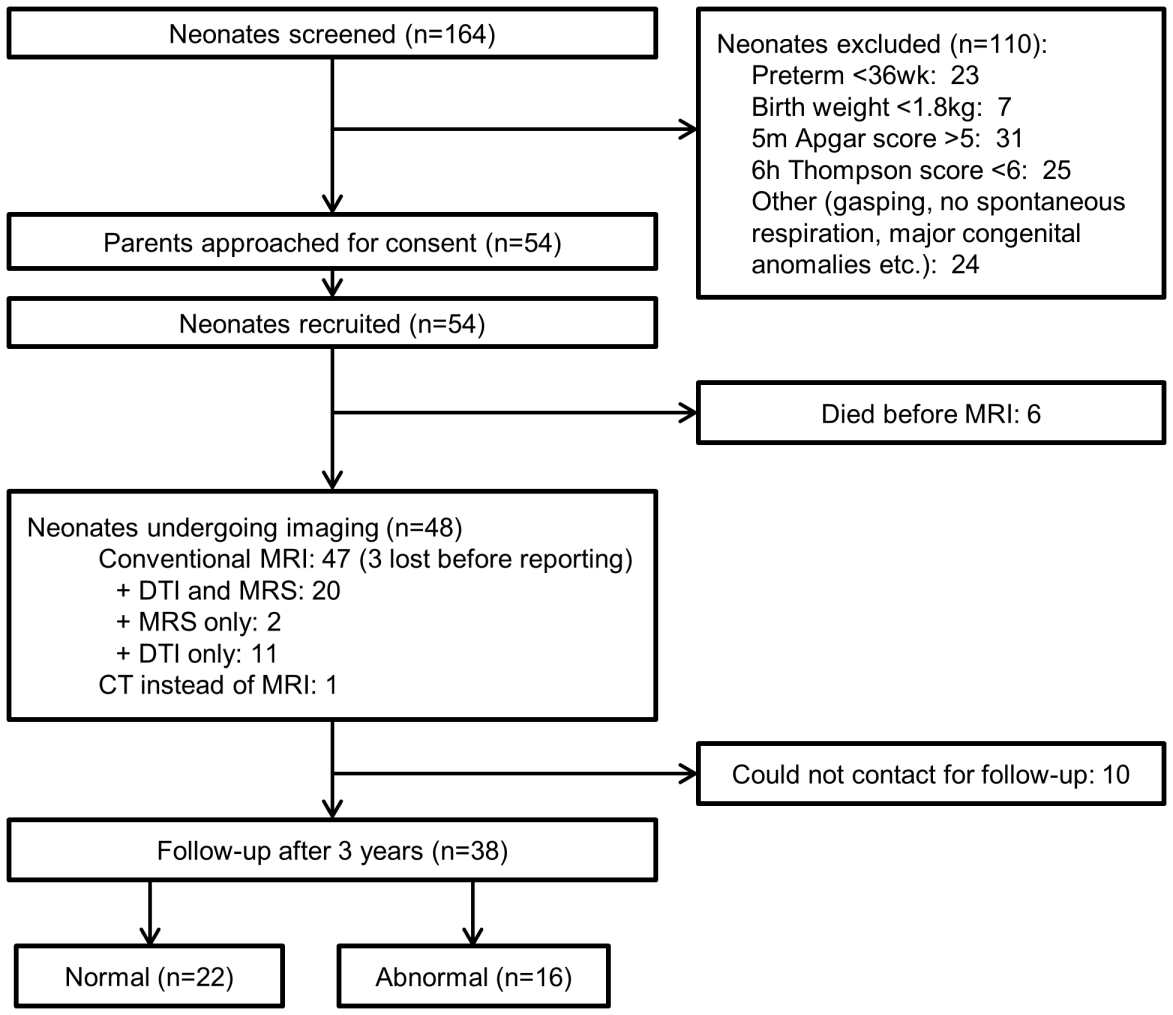
Study flow chart.

A total of 11,532 neonates were born at Government Medical College, Kozhikode during the 6 month recruitment period. Of these, 164 infants were eligibility screened subject to having a 5 min Apgar score <6 or requiring bag and mask/endotracheal tube resuscitation at birth: 54 met the inclusion criteria and were recruited into the study (Figure 1). Sarnat staging on day 3 showed mild encephalopathy in 30 infants (56%), moderate encephalopathy in 15 (28%) and severe encephalopathy in 6 (11%); 3 infants (6%) were not encephalopathic by this time. Demographic and biochemical data are given in Tables S1 and S2); along with blood pressure oxygen saturation and blood glucose levels (Figure S1). Four infants had significant hypoglycaemia, defined as a persistent low blood glucose (<2.6 mmol/l for ≥12 hours) as shown in Figure S2. Seventeen of the infants in the latter part of the study were randomised to cooling.

Thompson scores fell from days 1 to 4 in infants with mild encephalopathy (mean reduction of 4.7, 95% CI 4.0–5.4), but score decline was less marked in infants with moderate or severe encephalopathy (1.1, −0.7–2.8), as shown in Figure S3. The rate of this decrease was significantly higher in mild encephalopathy infants compared with moderate/severe infants (mean difference in reduction 3.6, −1.8–5.5, p<0.001). Six (11%) infants (4 with severe, 2 with moderate encephalopathy) died before discharge from hospital. In the remaining 48, neurological examination at discharge was abnormal in 22 (46%) of whom 8 (36%) had been cooled.

Thompson scores at age 6 hours poorly identified infants eligible for cooling as defined by Sarnat encephalopathy stage (moderate or severe) at age 3 days (Figure S4). Analysis of the receiver operator characteristic curve yielded an optimal cut-off of >7 (Youden index 0.47). Using this cut off, 28% of neonates considered eligible for cooling had mild encephalopathy and 22% of ineligible neonates had moderate encephalopathy on later Sarnat staging.

### Conventional MR Imaging

Of the 48 surviving infants, MR imaging was performed in 47 at mean (standard deviation) age 9.3 (3.6) days. One infant had computed X-ray tomography imaging only. Unfortunately, the MR images were lost in 3 cases before reporting and analysis for the study could be done. All the remaining MR images were of good enough quality for conventional reporting and without significant motion artefacts. Fourteen (32%) infants had small intracranial, mainly posterior fossa subdural bleeds not of long-term clinical significance.

No infants had evidence of antenatal/established brain injury on MR imaging, whilst all had some evidence of acute perinatal brain injury (Table 1). Forty (91%) had white matter injury; with 18 (41%) having mild changes, 16 (36%) moderate and 6 (14%) severe. Also common was involvement of the cortex, with 31 (70%) displaying abnormalities, though mostly mild. Only 12 (27%) infants showed any injury to the basal ganglia and thalami, mostly mild or moderate, and 5 (11%) displayed absence of the usual high signal intensity in the posterior limb of the internal capsule (PLIC) on T1 weighted imaging.

**Table 1:** Brain injury on conventional MR imaging.

Brain Region	Visual Interpretation	Normal†/mild neonatal encephalopathy (n = 29†)	Moderate‡/severe neonatal encephalopathy (n = 15‡)
Posterior limb of the internal capsule	0–Normal signal intensity	23 (79%)	9 (60%)
	1–Equivocal signal intensity	4 (14%)	3 (20%)
	2–Abnormal signal intensity	2 (7%)	3 (20%)
Basal ganglia and thalami	0–Normal	24 (83%)	8 (53%)
	1–Mild injury	2 (7%)	2 (13%)
	2–Moderate injury	3 (10%)	4 (27%)
	3–Severe injury	0 (0%)	1 (7%)
White matter	0–Normal	4 (14%)	0 (0%)
	1–Mild injury	11 (38%)	7 (47%)
	2–Moderate injury	12 (41%)	4 (27%)
	3–Severe injury	2 (7%)	4 (27%)
Cortex	0–Normal	9 (31%)	4 (27%)
	1–Mild injury	15 (52%)	6 (40%)
	2–Moderate injury	4 (14%)	3 (20%)
	3–Severe injury	1 (3%)	2 (13%)

Values are frequency (% of n).

† Three infants had no encephalopathy on day 3 Sarnat stage.

‡ 13 had moderate encephalopathy.

### DTI and Thalamic MR Spectroscopy

**Figure 2:**
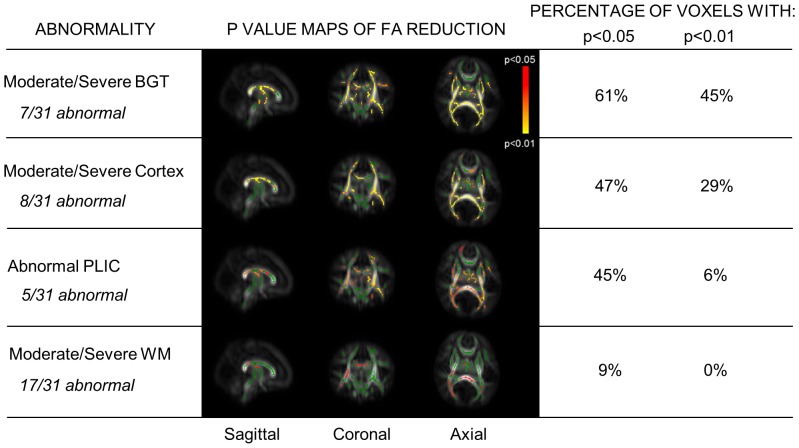
TBSS analysis of whole-brain white matter FA according to conventional MR imaging abnormalities. In each case FA is compared between the infants classified abnormal (by the criterion given), and the other infants in the cohort. Red-yellow pixels denote regions of white matter where FA values are different between groups with p<0.05–p<0.01, green pixels denote regions where p≥0.05.

**Figure 3:**
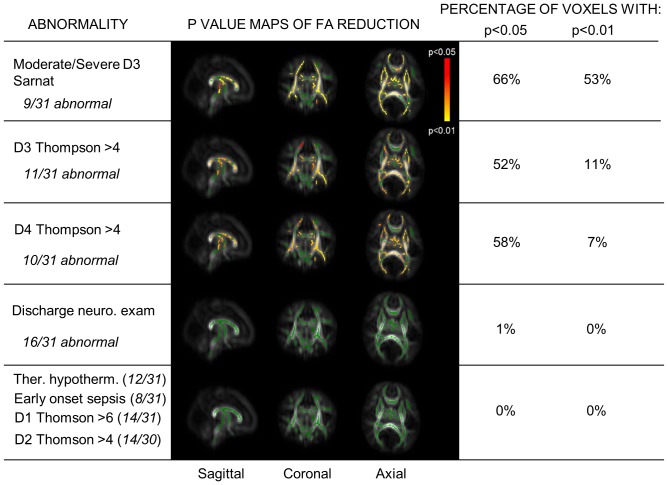
Whole-brain white matter FA according to perinatal clinical assessment. p value maps are displayed as described in Figure 2. Neuro = neurological; Ther. hypotherm. = therapeutic hypothermia.

A total of 31 infants had good quality DTI (including 5 with slice thicknesses between 5.5–6.5 mm) and 22 had MR spectroscopy data for analysis. Figure 2 shows the FA reductions according to conventional MR imaging grades. The proportion of the mean FA-skeleton voxels with significantly lower FA (p<0.05 and p<0.01) is shown in each case. Those infants with moderate/severe encephalopathy had globally reduced FA compared with the rest of the cohort (Figure 3). The same was seen for those with Thompson scores >4 at ages 3 and 4 days, but there was no significant relationship with Thompson score at age 2 days, or with scores >6 at age 6 hours. No significant FA changes were observed between cooled and normothermic infants, or between infants with and without signs of early onset sepsis.

Infants with moderate/severe basal ganglia/thalamic or cortical injury, or loss of the normal PLIC signal intensity displayed globally reduced FA compared to those without these findings. Although moderate/severe white matter injury was also associated with lower FA, the level of significance was lower (Figure 2).

Twenty-two infants underwent thalamic MR spectroscopy of whom 20 also had DTI. Using predefined cut-off thresholds [10], 2 of these infants (10%) had elevated lactate/N-acetylaspartate (≥0.29); 2 (10%) had elevated lactate/choline (≥0.25) and 3 (15%) had elevated lactate/creatine (≥0.39). Two infants (10%) had reduced N-acetylaspartate/choline (≤0.72), and none had abnormal N-acetylaspartate/creatine (≤1.20). Infants with abnormal N-acetylaspartate/choline had globally lower FA (Figure S5). There was no significant FA change for other metabolite ratio cut offs.

### Neurological Outcomes at Age 3½ years and Relation to Perinatal and Scan Data

Of the 48 survivors, 38 (79%) were seen for follow up (mean (standard deviation) assessment age of 3.4 (0.2) years). This included 24 (63%) with mild, 10 (26%) with moderate and 2 (5%) with severe neonatal encephalopathy (Table S3).

Abnormal outcome was found in 16 of the 38 infants seen; 3 had cerebral palsy (2 GMFSC level 1 and 1 with GMFCS level 5), 4 had a composite Bayley III cognitive score <85, 1 had abnormal vision, 9 had a fall in occipital-frontal circumference centile of >2 standard deviations and 3 had an ongoing need for anti-epileptics.

**Figure 4:**
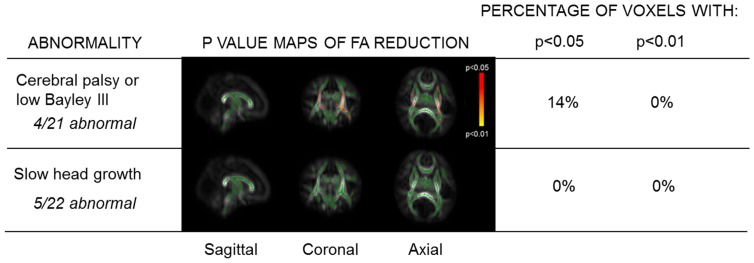
Whole-brain white matter FA according to outcome assessment aged 3½ years. p value maps are displayed as described in Figure 2, only using those with 3½ year outcome data for group-wise comparisons. Low Bayley III = infants with scores below predefined cut-offs for Bayley III (<82 for composite motor score, <85 for composite cognitive score); Slow head growth = isolated slow head growth (fall in head circumference centile from birth to follow-up of >2 standard deviations), with otherwise normal neurological examination and Bayley III scores.

An adverse outcome was found in 8 of 24 (33%) infants with mild encephalopathy, 5 of 10 (50%) with moderate, and 2 of 2 (100%) with severe encephalopathy (Tables 2 and 3). Two of the infants with mild encephalopathy who had adverse outcomes (1 with cerebral palsy and 1 with slow head growth) also both had mild prolonged perinatal hypoglycaemia (Figure S2). There was a significant FA reduction in neonates with cerebral palsy or low Bayley III scores compared to the group of neonates with normal outcomes (Figure 4). There was no significant FA difference in those with slow head growth compared to this normal outcome group. MRI scoring was most accurate in the basal ganglia/thalami and PLIC for identifying poor neurodevelopmental outcomes at early childhood (Figure S6).

**Table 2:** Characteristics of children with adverse early childhood outcomes.

Patient	Day 3 Sarnat	MRI abnormality scores (BG,T,BGT,WM,Cortex)	Hypogly-caemia	CP	GMFCS level	AE	Discharge Neurology	Bayley III (M,C,L)	Birth/3½ year HC centile	HC Z-Score Change	Hearing at 3½ years	Vision at 3½ years
1	Normal	0, 0, 0, 1, 0	No	No	0	No	Normal	130, 95, 105	83/0.2	−3.85	Normal	Normal
2	Mild	0, na, 0, 3, 3	Yes	Yes	1	Yes	Normal	na, na, na	12/<0.1	−2.93	Normal	Abnormal†
3	Mild	0, 0, 0, 0, 1	No	No	0	Yes	Normal	107, 90, 106	12/9	−0.22	Normal	Normal
4	Mild	0, 0, 0, 1, 0	No	No	0	No	Normal	118, 95, 100	54/0.1	−3.32	Normal	Normal
5*	Mild	1, 1, 1, 1, 1	No	No	0	No	Normal	124, 95, 103	6/<0.1	−2.38	na	na
6	Mild	0, 0, 0, 1, 1	No	No	0	No	Normal	100, 90, 91	66/0.1	−3.56	Normal	Normal
7*	Mild	2, 2, 2, 2, 2	No	No	0	No	Normal	110, 90, 100	23/<0.1	−2.90	Normal	Normal
8	Mild	0, 0, 0, 2, 1	No	No	0	No	Normal	85, 75, 94	12/12	−0.03	Normal	Normal
9	Mild	0, 0, 0, 3, 2	Yes	No	0	No	Normal	115, 90, 97	89/0.5	−3.78	Normal	Normal
10	Moderate	0, 0, 0, 1, 1	No	No	0	No	Abnormal	103, 95, 91	79/4	−2.60	Normal	Normal
11	Moderate	0, 1, 1, 1, 0	No	No	0	No	Abnormal	124, 80, 103	12/na	na	na	na
12*	Moderate	1, 3, 2, 1, 1	No	Yes	1	na	Abnormal	na, na, na	66/na	na	Normal	Normal
13	Moderate	2, 2, 2, 3, 2	No	No	0	No	Abnormal	85, 80, 94	22/3	−1.12	Normal	Normal
14*	Moderate	3, 3, 3, 3, 2	No	Yes	5	Yes	Abnormal	na, na, na	23/na	na	Normal	Normal
15	Severe	2, 1, 2, 2, 3	No	No	0	No	Abnormal	110, 95, 103	22/0.2	−2.08	Normal	Normal
16	Severe	0, 0, 0, 1, 0	No	No	0	No	Abnormal	94, 75, 79	12/7	−0.30	Normal	Normal

BG = basal ganglia; T = thalami; BGT = composite basal ganglia and thalami, WM = white matter; GMFCS = Gross Motor Function Classification System; CP = cerebral palsy at follow-up; AE = anti-epileptics at follow-up; M = composite motor score; C = composite cognitive score; L = composite language score; HC = head circumference; na = not available/applicable. MRI scoring system described in Table 3, under ‘Visual Interpretation’.

*Underwent therapeutic hypothermia.

† Requiring visual aids.

**Table 3:** Early childhood outcomes of infants according to Sarnat neonatal encephalopathy staging.

Outcome	Mild encephalopathy(n = 24)	Moderate encephalopathy (n = 12)	Severe encephalopathy (n = 6)
Died	0 (0%)	2 (17%)	4 (67%)
Survival with normal outcome* at 3½ years	16 (67%)	5 (42%)	0 (0%)

Values are frequency (% of n);

*Normal outcome defined as Bayley III cognitive score ≥85 and motor composite score ≥82; with normal head growth; normal neurological examination; normal vision and hearing; and no ongoing seizures at 3½ years.

## Discussion

Although the nature of brain injury and early childhood outcomes in neonatal encephalopathy is well studied in high-income countries, there is very limited data from low and middle-income countries. In this prospective cohort study from a low and middle-income country, we report several important observations in the nature of brain injury that may be useful in developing neuroprotective strategies for this population. Most infants studied (91%) had some change in white matter signal on conventional MR imaging; 41% had mild abnormality, 36% moderate and 14% severe based on a previously validated MR imaging scoring system [18]. Only 27% had any basal ganglia/thalamic injury and this was mild or moderate though may have been more severe in the 6 infants who died but were not imaged. Whole-brain white matter FA was associated with reduced MR spectroscopy N-acetylaspartate/choline, cortical and basal ganglia/thalamic injury and loss of the normal PLIC signal intensity, but not with visual assessment of white matter injury on conventional MR imaging. Conversely, the severity of the Thompson encephalopathy score at age <6 hours did not parallel the severity of brain injury assessed on MRI, nor was it useful in the early identification of potential candidates (i.e. moderate or severe encephalopathy) for neuroprotective therapies. However, whole-brain white matter FA was strongly associated with Sarnat encephalopathy staging. More importantly, the brain injury seen in this prospective cohort appears to be of perinatal origin, and hence is potentially amenable to cooling therapy; none of the infants had MRI evidence of established brain injury clearly preceding the intrapartum period.

Of the 54 infants recruited, 6 (11%) died before discharge. Thirty-eight of the remaining 48 infants had early childhood outcome evaluations. Adverse outcomes were seen in 16/38 (42%) infants followed until age 3½ years. This included 3 (8%) children with cerebral palsy, 9 (24%) with slow head growth and 4 (11%) with low scores on the Bayley III assessment [28]. Furthermore, in infants with mild neonatal encephalopathy, 8 (27%) had adverse outcomes. Prolonged though mild hypoglycaemia during the first 90 hours after birth was recorded in 2 (25%) of these infants.

The incidence of adverse outcomes at age 3½ years after mild encephalopathy in our study is higher than that reported from high-income countries, but comparable to data from Nepal [29], where adverse outcome was reported in 9 (26%) of the 35 infants with mild encephalopathy followed up to 1 year of age. As no blood or imaging investigations were performed, the exact cause of higher incidence of adverse outcomes in infants with mild encephalopathy was unclear in that study. In addition, we included slow head growth as an adverse outcome unlike the previous studies, which might explain our higher rate of unfavourable outcome. We also chose to use higher Bayley III cut-offs in comparison to other studies using Bayley II, due to the apparent discrepancies in normal ranges [28]. We considered slow head growth with normal Bayley III scores and normal neurological examination at 3½ years as an adverse outcome due to the potential association of learning difficulties, and other behavioural problems in infants with slow head growth when followed up to school age [30], [31]. Further follow up of these infants may be required to examine the relationships between our findings and intelligence quotient and any behavioural problems.

Our use of tensor-based registration in DTI-TK has minimised the amount of bias in choosing a representative template for inter-subject analysis of white matter FA. Where comorbidity groupings were associated with dramatic FA changes, the tensor-based-registration method did not necessarily translate into higher overall t-statistics than when using FA-based registration [32], but there were noticeable reductions in significance for comparisons which had yielded borderline significance with the FA-based method (e.g. thalamic lactate/N-acetylaspartate, white matter appearance on conventional MR imaging, discharge neurology). While the TBSS method incorporates a correction for multiple voxel-wise comparisons within each group-wise test (‘threshold-free cluster enhancement [21]’), the range of tests performed was for exploratory analysis, and so should be interpreted with care due to the reduced significance of each result. Each test we performed has therefore been included to ensure transparency.

The high incidence of white matter abnormality seen on MRI scan in this cohort is in sharp contrast to the predominantly basal ganglia/thalamic pattern of lesions seen in high-income countries, which are highly associated with poor outcomes [18], [33]–[35]. This difference in injury pattern may be somewhat exaggerated since 6 infants died before MR imaging, and in these more severe cases basal ganglia/thalamic injury is often seen. Strong association of whole brain white matter FA with thalamic N-acetylaspartate/choline, a marker of cell membrane injury, indicates a close relationship between thalamic metabolic perturbations and white matter microstructure. Given this, the relatively weak dependence of white matter FA on white matter MR imaging appearance was unexpected, and suggests that visual MR imaging interpretation of this injury may be subjective or transient – however most of the white matter change seen in this group was mild or moderate and been shown in studies of encephalopathy in high-income countries to be associated with good outcomes [35]. The dependence on basal ganglia/thalamic injury agrees with others [18], [34], [35] who associated such a relationship with poorer neurodevelopmental outcomes. Indeed, 18 of 33 (55%) infants with white matter injury and follow-up data went on to have normal outcomes at age 3½ years; in contrast to the 4 of 11 (36%) infants with any basal ganglia/thalami injury who had normal outcomes. Although we collected composite language scores in the Bayley III assessment, these were not analysed due to the subjectivity of their non-English application. Since the assessment was performed close to the upper age range for Bayley III, our results may suffer from a ‘ceiling effect’ but it is unlikely that children would fall below a score of 85 for this reason.

Given the increasing understanding of longer-term neurodevelopment problems arising from perinatal white matter injury, such abnormalities may not be apparent at early follow-up, in agreement with the findings of de Vries and others [36], [37]. This would also explain the poor agreement between FA and the neurological discharge examination.

Within 6 hours of birth, Thompson scores [14] are poor at identifying infants eligible for cooling, as illustrated in the Supporting Information (Figure S4), and we suggest should not be used for this purpose. As the cut-off score increases, fewer neonates with mild neonatal encephalopathy would be chosen for cooling but more moderate/severe cases would be considered ineligible. Even at a score cut-off of 9, 10% of the cooled neonates would have mild neonatal encephalopathy, and 27% of uncooled neonates would have moderate/severe encephalopathy as judged by the day 3 Sarnat score. This is reinforced by the lack of FA change when assessing the cohort according to Thompson score within the first 2 days after birth. This may be due to the non-physiological allocation of equal score to any abnormality in the Thompson score, as opposed to more physiological categorisation used in the recently validated NICHD encephalopathy scoring system [38], and hence the latter may be a better clinical criterion for identifying potential candidates for cooling.

No significant white matter microstructural changes were associated with therapeutic hypothermia. This observation is not entirely unexpected, as the study was not intended to examine the therapeutic effect of hypothermia and the infants who received cooling were not adequately matched with normothermic infants in terms of other clinical morbidities, as would be the case in a randomised controlled trial (Table S4). Nevertheless, adequately powered clinical trials would be required to evaluate cooling in low- and middle-income countries, as it remains unclear whether cooling therapy would be have a similar safety and efficacy profile outside the setting of a high-income country tertiary neonatal intensive care unit [39]. As a tentative guide for future studies, the specificity and sensitivity of MRI in identifying adverse outcomes in this cohort is given in Supporting Information (Table S5).

Our study is limited by the absence of a sufficient control group, as white matter microstructural changes have been compared between sub-groups of the same cohort. We were unable to image infants who died early hence the imaging patterns described are limited to surviving infants. In addition, we were unable to acquire follow-up data on 19% of the infants; hence, we were limited in examining the overall neurodevelopment of the cohort. As the early childhood follow-up was originally unfunded, we were unable to maintain regular contact with the families after hospital discharge, and could only contact the parents if their contact details were unchanged. All the parents contacted attended follow-up assessments and it is likely that high follow-up rates can be obtained if regular family contact is maintained in future studies. Reassuringly, no systematic differences were seen in the clinical characteristics or brain injury in the infants lost to follow-up, and those who attended 3½ year follow-up, suggesting the outcome data may be generalised to the whole cohort.

Due to the lack of available data on the application of MRS in this population, we have applied cut-off values derived from studies in high income countries. By applying the same acquisition scheme as used in the UK, we have minimised the effect of any systematic differences in protocol. However, it may be the case that altered cut-off values may identify adverse outcomes more optimally in this setting.

The strength of the study is in the use of a combination of novel MR biomarkers to uniquely describe different facets of brain injury in this population. The advantage of TBSS is its ability to detect significant white matter microstructural differences even between small groups, enabling meaningful inferences with limited numbers of subjects. In addition, the bias in using a standard ‘most representative subject’ template in TBSS has been overcome by adapting standard post-processing techniques with DTI-TK [40], [41].

In summary, the population co-morbidities, patterns of brain injury and early childhood outcomes in neonatal encephalopathy in this low- and middle-income country cohort have important characteristics. White matter injury was common, though mainly mild, although injury to the basal ganglia and thalami, even though only mild or moderate, was most predictive of abnormal neurological outcomes. White matter microstructural abnormality on TBSS was most significantly associated with injury to the basal ganglia and thalami, thalamic N-acetylaspartate/choline and with Sarnat staged encephalopathy severity but not with early Thompson encephalopathy score. Finally, the lack of established brain injury in this cohort indicated that lesions were likely of perinatal in origin, and therefore potentially treatable. These data support rigorous evaluation of rescue hypothermic neuroprotection in low- and middle-income countries in clinical trials.

## Supporting Information

Figure S1 **Blood glucose, oxygen saturation (SaO_2_) and blood pressure (mean and standard deviation error bar) in all infants in the first 4 days after birth.** Norm/Mild = normal/mild Sarnat encephalopathy stage at 3 days after birth; Mod/Sev = moderate/severe Sarnat encephalopathy stage at 3 days after birth.
(TIF)

Figure S2 **Individual blood glucose measurements in the 4 hypoglycaemic infants in the first 4 days after birth according to 3½ year outcome (Unknown = unpresented for 3½ year assessment).**
(TIF)

Figure S3 **Mean (standard deviation) Thompson scores at ages up to 4 days in infants grouped according to Sarnat neonatal encephalopathy (NE) stage assessed at 3 days after birth.**
(TIF)

Figure S4 **Use of the 6-hour Thompson score as an inclusion criterion for cooling therapy compared to the Sarnat encephalopathy stage (normal, mild, moderate or severe) at 3 days after birth.** Sensitivity and specificity: infants with moderate/severe Sarnat stage treated as disease positive, normal/mild stage as disease negative.
(TIF)

Figure S5 **Whole-brain white matter FA according to MR spectroscopy assessment.** p value maps are displayed as described in Figure 2, only using those with MR spectroscopy data for group-wise comparisons. NAA = N-acetylaspartate; Lac = lactate; Cho = choline-containing compounds; Cr = total creatine.
(TIF)

Figure S6 **Conventional MR imaging (MRI) scores across infants with different 3½ year outcomes.** The solid horizontal line indicates median score for each group. T = thalami; BG = basal ganglia; CP = infants with cerebral palsy; Low BIII = infants with scores below predefined cut-offs for Bayley III (<82 for composite motor score, <85 for composite cognitive score); Slow HG = isolated slow head growth (fall in head circumference centile from birth to follow-up of >2 standard deviations) with otherwise normal neurological examination and Bayley III scores; Normal = infants with normal outcome at follow-up.
(TIF)

Table S1 **Clinical characteristics.** Values are mean (standard deviation) or proportion (%) unless otherwise indicated. †Clinical sepsis with elevated C-reactive protein with or without positive blood culture, requiring antibiotic treatment, within three days of birth; CI = confidence interval. *Indicates difference between groups with p<0.05.
(DOCX)

Table S2 **Biochemical characteristics.** Values are mean (standard deviation) unless otherwise indicated. ^†^Denotes skewed distributions where median (IQR) is reported, along with the difference in medians (p value of Mann-Whitney U test). *Indicates difference between group means with p<0.05. CI = confidence interval; WBC = white blood cells; SGPT = serum glutamic-pyruvic transaminase; PT = prothrombin time; APTT = activated partial thromboplastin time.
(DOCX)

Table S3 **Clinical features and brain injury of surviving infants who attended 3½ year** **follow-up or were lost to follow-up.** Values are proportion (%) unless otherwise indicated. CI = confidence interval; WM = white matter; BGT = basal ganglia and thalami; PLIC = posterior limb of the internal capsule.
(DOCX)

Table S4 **Characteristics and outcomes of neonatal encephalopathy for infants undergoing whole-body therapeutic hypothermia or normothermia.** Values are mean (standard deviation) or proportion (%). ^†^Sepsis =  Clinical sepsis with elevated C-reactive protein with or without positive blood culture within three days of birth. ^‡^Abnormal outcome = cerebral palsy; visual or hearing impairment; evidence of seizures or use of anti-epileptic medication at age 3½ years; slowed head growth; or a composite motor score <82 or composite cognitive score <85 on Bayley III.
(DOCX)

Table S5 **Sensitivity and specificity of conventional MR imaging in identifying infants with cerebral palsy or low Bayley III scores (<82 for composite motor, <85 for composite cognitive) at 3½ years.**
(DOCX)
