# Correction: Neonatal Encephalopathic Cerebral Injury in South India Assessed by Perinatal Magnetic Resonance Biomarkers and Early Childhood Neurodevelopmental Outcome

**DOI:** 10.1371/journal.pone.0092526

**Published:** 2014-03-10

**Authors:** The PLOS ONE Staff

The affiliations of the 12^th^ and 15^th^ authors are incorrect. Angie Wade and Betty Hutchon are affiliated with the following institution: Institute of Child Health, University College London, London, United Kingdom.

## References

[1] Lally PJ, Price DL, Pauliah SS, Bainbridge A, Kurien J, et al (2014) Neonatal Encephalopathic Cerebral Injury in South India Assessed by Perinatal Magnetic Resonance Biomarkers and Early Childhood Neurodevelopmental Outcome. PLoS ONE 9(2): e87874 doi:10.1371/journal.pone.0087874 24505327 10.1371/journal.pone.0087874 PMC3914890

