# Supplementary figures and images for: Neonatal Encephalopathic Cerebral Injury in South India Assessed by Perinatal Magnetic Resonance Biomarkers and Early Childhood Neurodevelopmental Outcome
Source: PLoS One. 2014 Feb 5;9(2):e87874. doi: 10.1371/journal.pone.0087874 (PMC3914890; doi:10.1371/journal.pone.0087874)

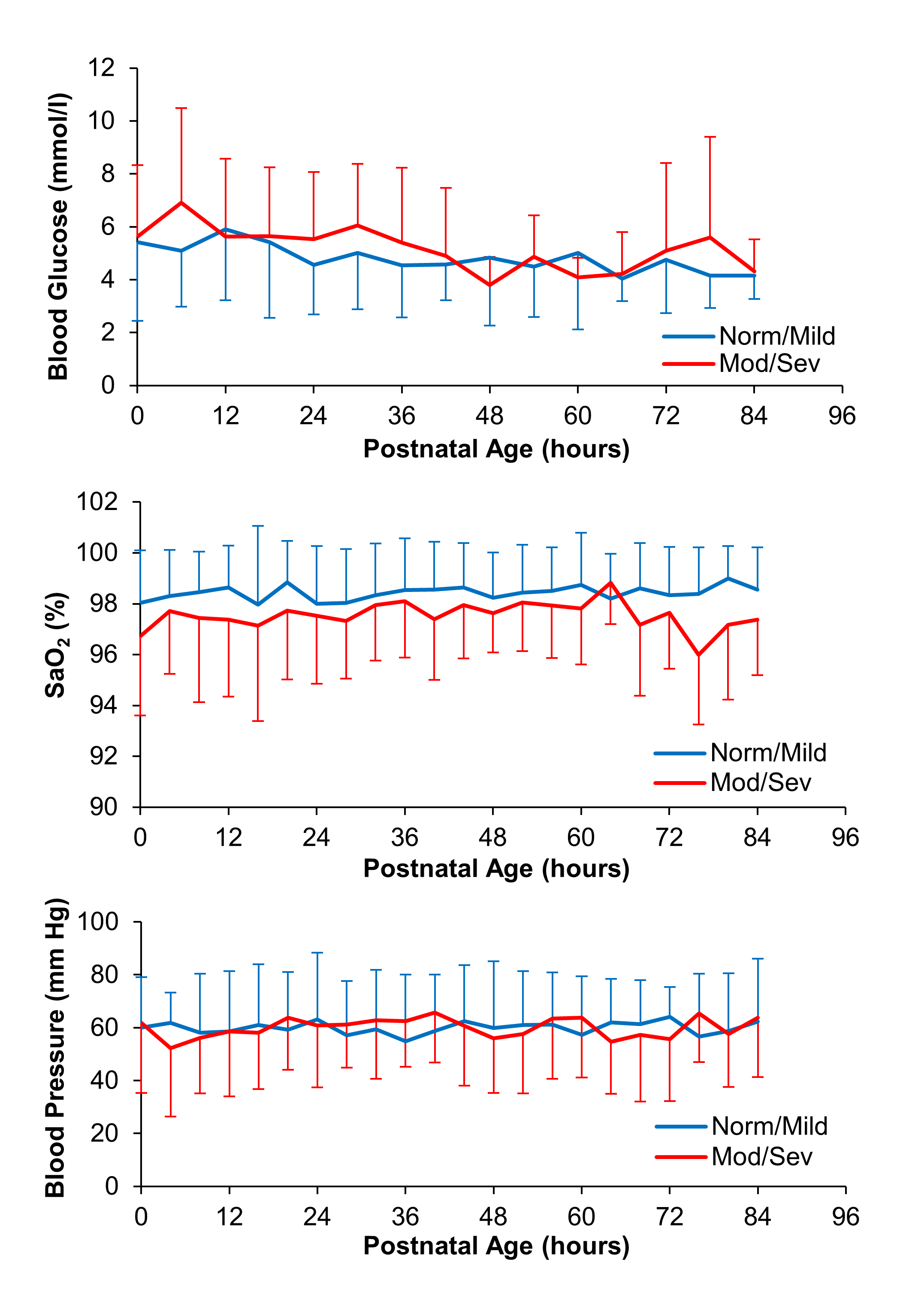

Supplement: Figure S1 — Blood glucose, oxygen saturation (SaO2) and blood pressure (mean and standard deviation error bar) in all infants in the first 4 days after birth. Norm/Mild = normal/mild Sarnat encephalopathy stage at 3 days after birth; Mod/Sev = moderate/severe Sarnat encephalopathy stage at 3 days after birth. (TIF) [file pone.0087874.s001.tif]

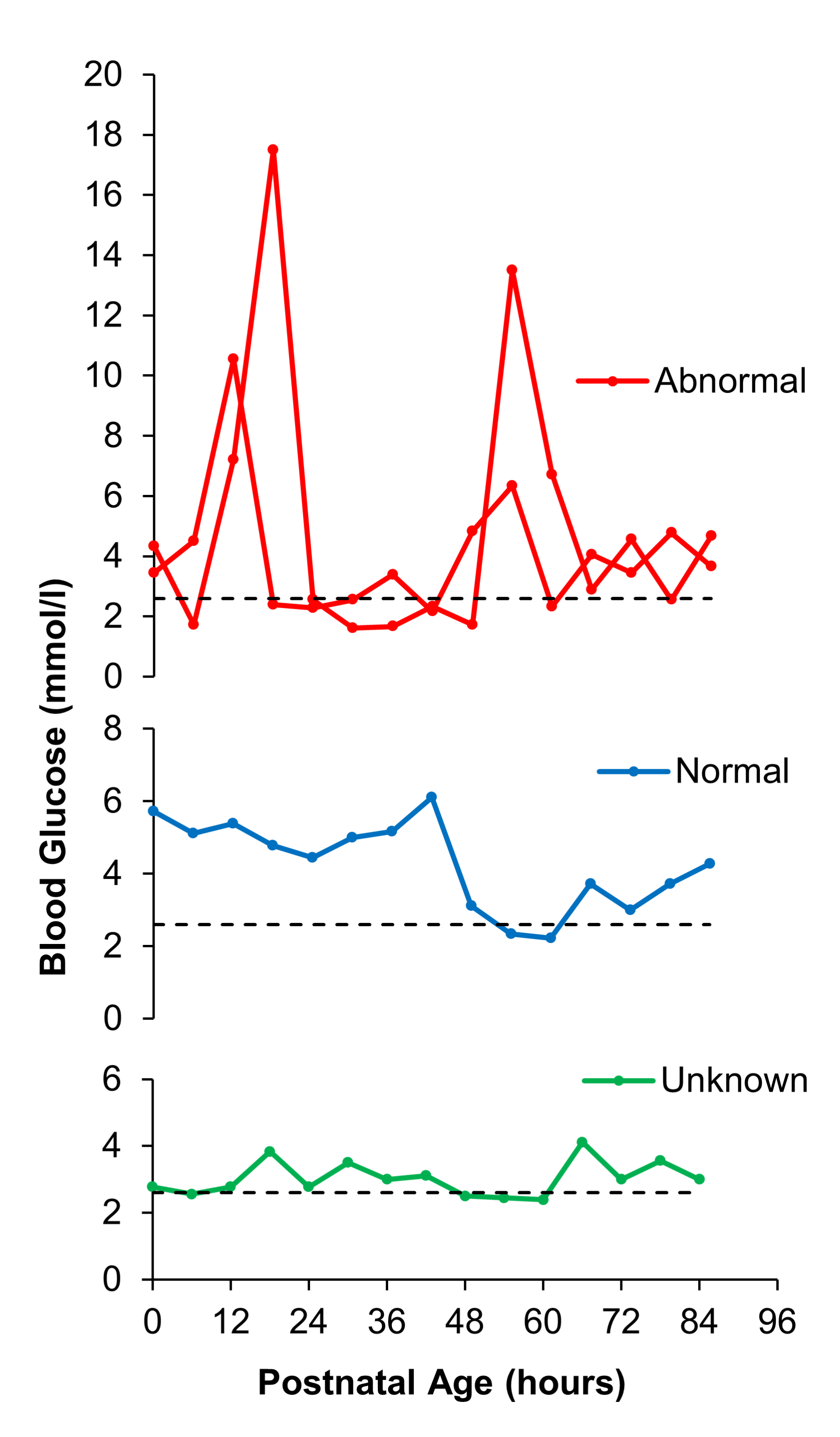

Supplement: Figure S2 — Individual blood glucose measurements in the 4 hypoglycaemic infants in the first 4 days after birth according to 3½ year outcome (Unknown = unpresented for 3½ year assessment). (TIF) [file pone.0087874.s002.tif]

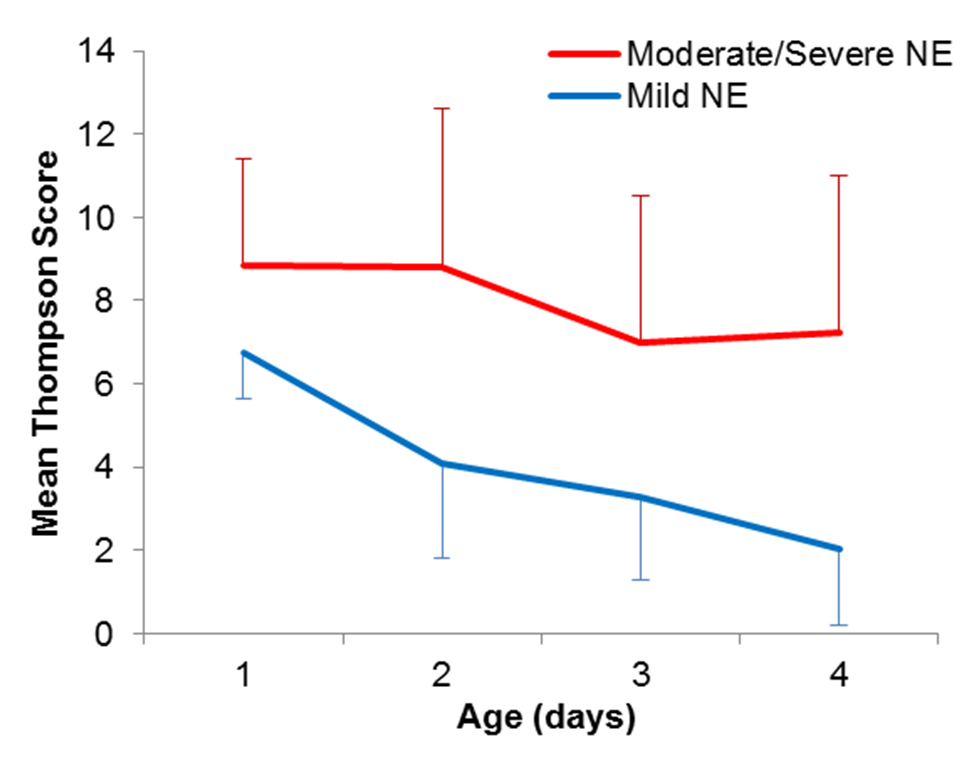

Supplement: Figure S3 — Mean (standard deviation) Thompson scores at ages up to 4 days in infants grouped according to Sarnat neonatal encephalopathy (NE) stage assessed at 3 days after birth. (TIF) [file pone.0087874.s003.tif]

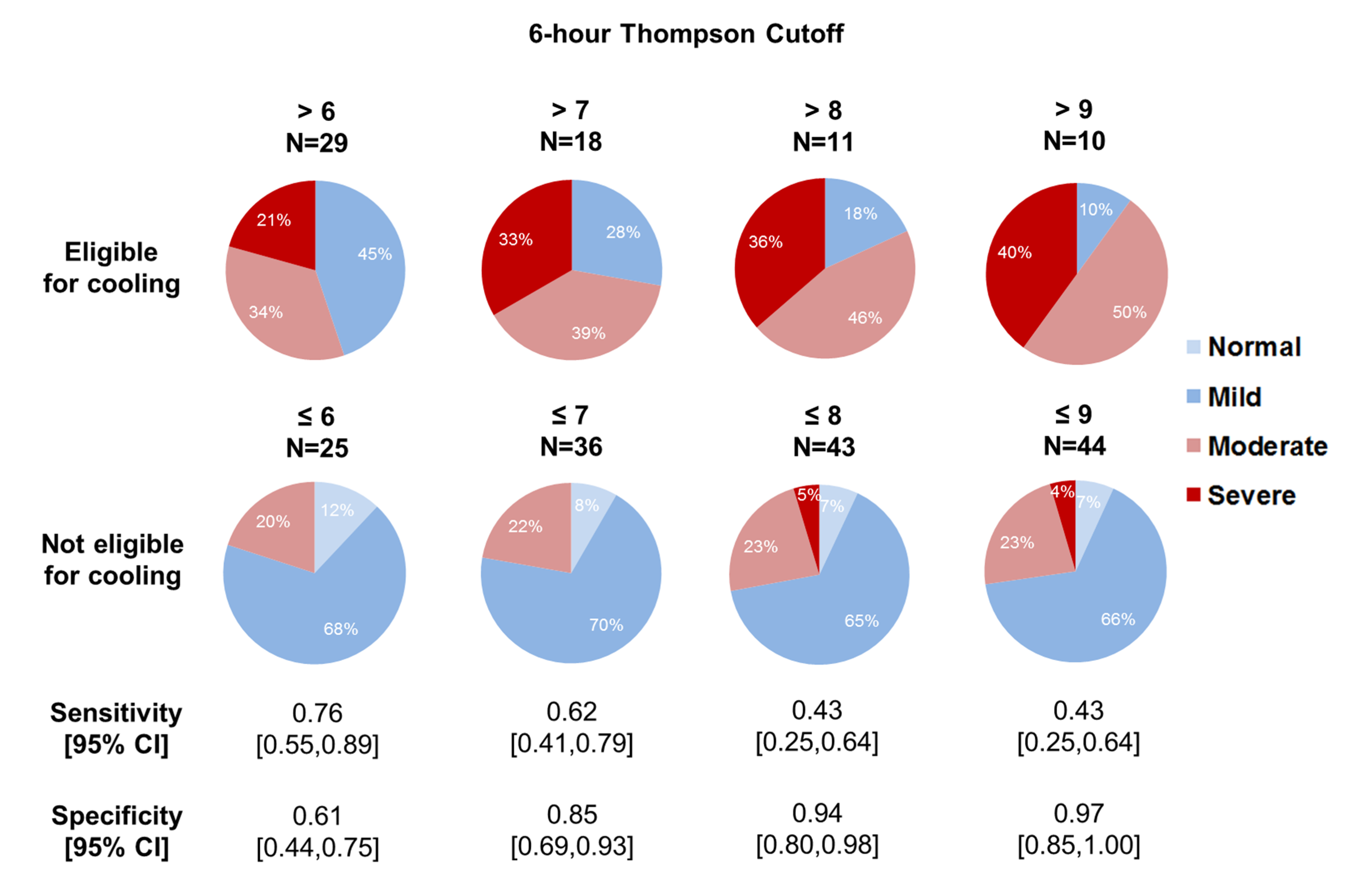

Supplement: Figure S4 — Use of the 6-hour Thompson score as an inclusion criterion for cooling therapy compared to the Sarnat encephalopathy stage (normal, mild, moderate or severe) at 3 days after birth. Sensitivity and specificity: infants with moderate/severe Sarnat stage treated as disease positive, normal/mild stage as disease negative. (TIF) [file pone.0087874.s004.tif]

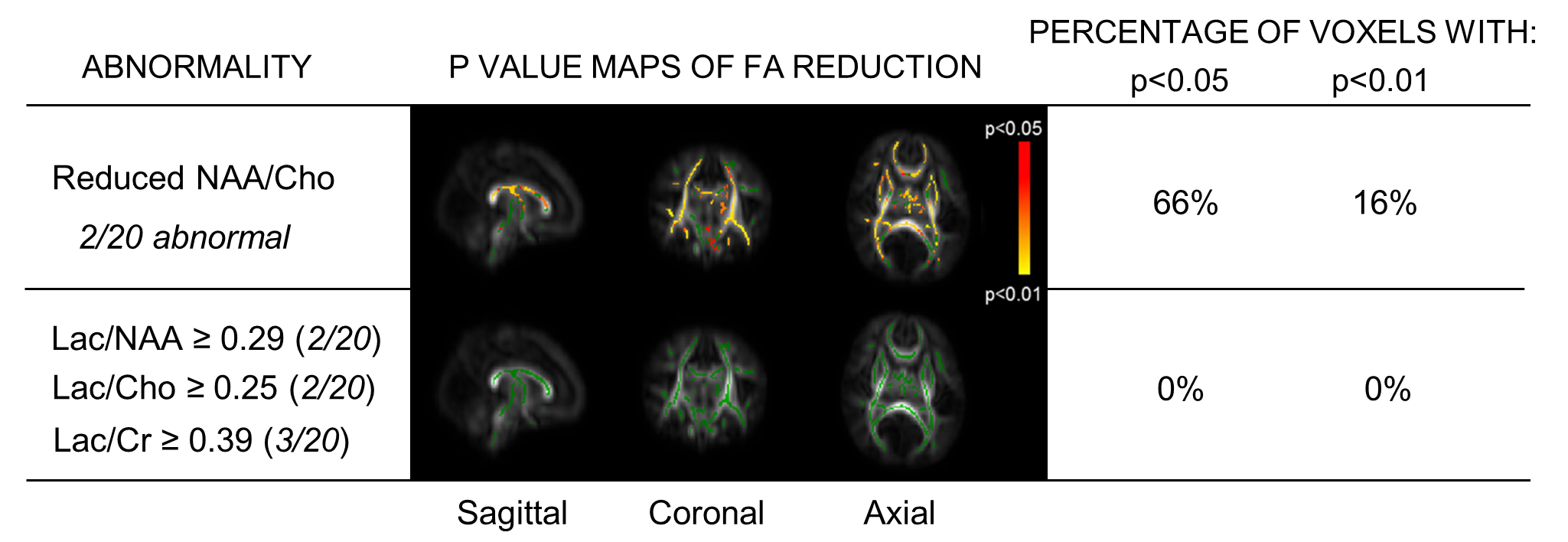

Supplement: Figure S5 — Whole-brain white matter FA according to MR spectroscopy assessment. p value maps are displayed as described in Figure 2, only using those with MR spectroscopy data for group-wise comparisons. NAA = N-acetylaspartate; Lac = lactate; Cho = choline-containing compounds; Cr = total creatine. (TIF) [file pone.0087874.s005.tif]

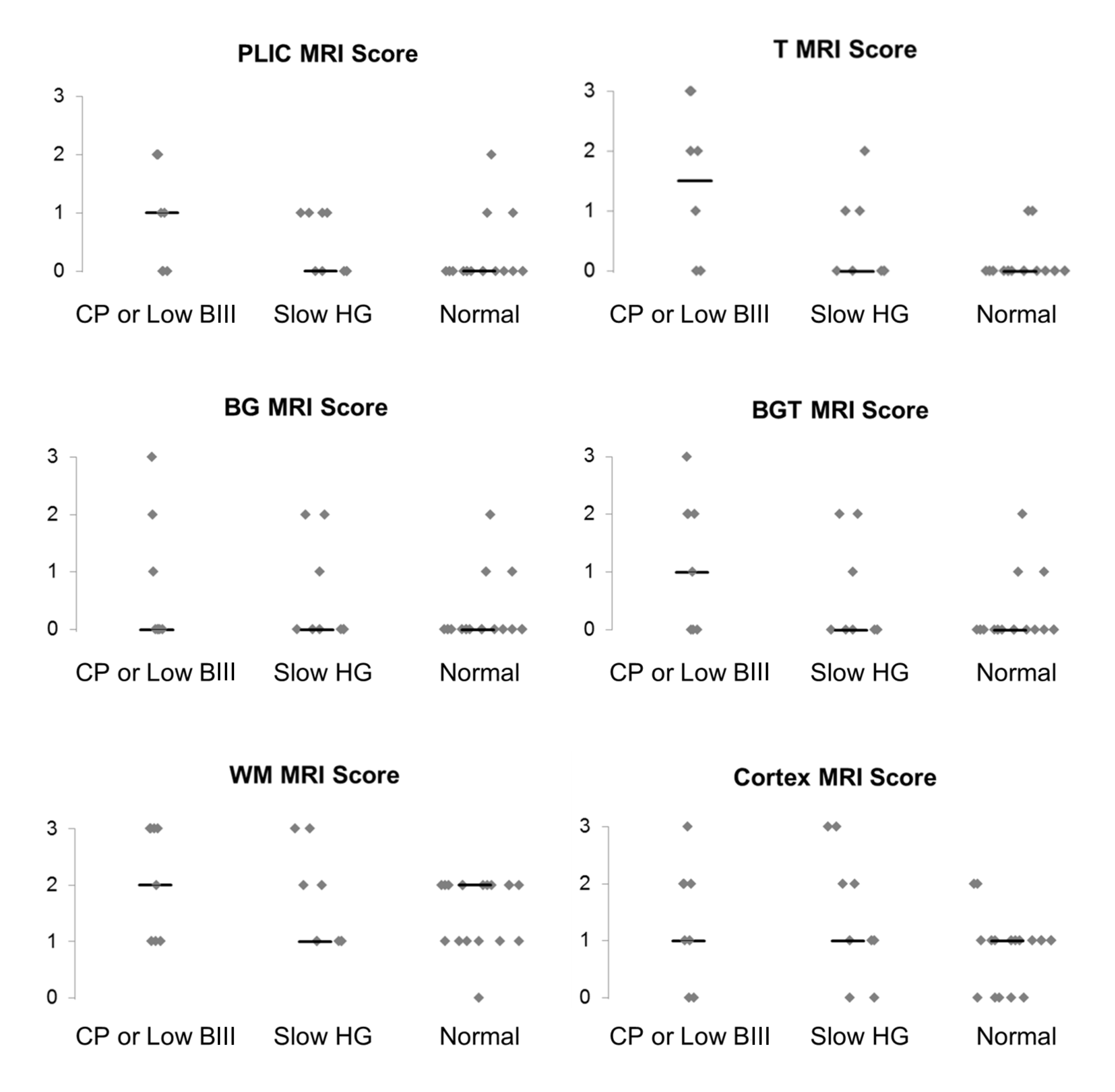

Supplement: Figure S6 — Conventional MR imaging (MRI) scores across infants with different 3½ year outcomes. The solid horizontal line indicates median score for each group. T = thalami; BG = basal ganglia; CP = infants with cerebral palsy; Low BIII = infants with scores below predefined cut-offs for Bayley III (<82 for composite motor score, <85 for composite cognitive score); Slow HG = isolated slow head growth (fall in head circumference centile from birth to follow-up of >2 standard deviations) with otherwise normal neurological examination and Bayley III scores; Normal = infants with normal outcome at follow-up. (TIF) [file pone.0087874.s006.tif]
