# Supplementary material for: Neonatal Encephalopathic Cerebral Injury in South India Assessed by Perinatal Magnetic Resonance Biomarkers and Early Childhood Neurodevelopmental Outcome
Source: PLoS One. 2014 Feb 5;9(2):e87874. doi: 10.1371/journal.pone.0087874 (PMC3914890; doi:10.1371/journal.pone.0087874)
Supplement: Table S1 — Clinical characteristics. Values are mean (standard deviation) or proportion (%) unless otherwise indicated. †Clinical sepsis with elevated C-reactive protein with or without positive blood culture, requiring antibiotic treatment, within three days of birth; CI = confidence interval. *Indicates difference between groups with p<0.05. (DOCX) [file pone.0087874.s007.docx]

Table S1

| Clinical Characteristic | Normal/Mild neonatal encephalopathy (n=33) | Moderate/Severe neonatal encephalopathy (n=21) | Mean Difference (95% CI) |
| --- | --- | --- | --- |
| Number of antenatal visits | 6.3 (1.4) | 6.8 (1.8) | +0.5 (-0.4,1.4) |
| Emergency Caesarean sections | 5/32 (16%) | 3/20 (15%) | -1% (-20%,22%) |
| Birth weight (g) | 2943 (403) | 2818 (386) | -125 (-347,98) |
| Gestation at birth (weeks + days) | 38+1 (0+9) | 38+3 (0+7) | +2 (-3,7) |
| Head circumference at birth (cm) | 33.8 (1.1) | 33.7 (0.8) | -0.1 (-0.6,0.5) |
| Apgar score 1 min | 1.9 (1.0) | 2.0 (0.8) | +0.1 (-0.5,0.7) |
| Apgar score 5 min | 4.3 (1.0) | 4.4 (0.8) | +0.1 (-0.5,0.7) |
| Apgar score 10 min | 7.0 (1.5) | 6.5 (1.2) | -0.5 (-1.6,0.6) |
| Early onset sepsis† | 11/33 (33%) | 5/21 (24%) | -9% (-33%,19%) |
| Blood stream infection | 4/33 (15%) | 2/21 (11%) | -4% (-23%,20%) |
| Death before discharge | 0/33 (0%) | 6/21 (29%) | +29% (9%,53%)* |
| Abnormal discharge neurology | 8/33 (24%) | 14/21 (67%) | +43% (13%,66%)* |
