# Supplementary material for: Neonatal Encephalopathic Cerebral Injury in South India Assessed by Perinatal Magnetic Resonance Biomarkers and Early Childhood Neurodevelopmental Outcome
Source: PLoS One. 2014 Feb 5;9(2):e87874. doi: 10.1371/journal.pone.0087874 (PMC3914890; doi:10.1371/journal.pone.0087874)
Supplement: Table S2 — Biochemical characteristics. Values are mean (standard deviation) unless otherwise indicated. †Denotes skewed distributions where median (IQR) is reported, along with the difference in medians (p value of Mann-Whitney U test). *Indicates difference between group means with p<0.05. CI = confidence interval; WBC = white blood cells; SGPT = serum glutamic-pyruvic transaminase; PT = prothrombin time; APTT = activated partial thromboplastin time. (DOCX) [file pone.0087874.s008.docx]

Table S2

| Age | Measurement | Normal/Mild neonatal encephalopathy (n=33) | Moderate/Severe neonatal encephalopathy (n=21) | Difference in means or medians (95% CI or p value) |
| --- | --- | --- | --- | --- |
| <6 hours | Haemoglobin (g/dl) | 16.7 (1.7) | 16.1 (2.1) | -0.6 (-1.6,0.4) |
|  | WBC count (109/l) | 21.4 (8.2) | 24.3 (10.8) | +2.9 (-3.0,8.7) |
|  | Platelet count (105/µl) | 2.2 (0.6) | 2.9 (1.9) | +0.7 (-0.2,1.6) |
|  | ^†^C-reactive protein (mg/l) | 1.5 (1.7) | 1.9 (3.8) | +0.4 (p=0.37) |
|  | Urea (mg/dl) | 18.8 (5.1) | 18.3 (7.3) | -0.5 (-4.2,3.3) |
|  | ^†^Creatinine (mg/dl) | 0.60 (0.10) | 0.60 (0.10) | 0.0 (p=0.92) |
|  | Sodium (mEq/l) | 137.5 (2.9) | 136.3 (3.9) | -1.2 (-3.3,1.0) |
|  | Potassium (mEq/l) | 4.8 (1.0) | 4.9 (0.7) | +0.1 (-0.4,0.7) |
|  | ^†^SGPT (U/l) | 29.0 (26.0) | 53.0 (69.5) | +24.0 (p=0.11) |
|  | ^†^PT (seconds) | 17.5 (4.3) | 18.0 (3.5) | +0.5 (p=0.56) |
|  | APTT (seconds) | 43.2 (12.3) | 38.1 (12.5) | -5.1 (-12.8,2.7) |
| 72-90 hours | ^†^Haemoglobin (g/dl) | 16.3 (2.3) | 16.3 (3.7) | 0.0 (p=1.00) |
|  | ^†^WBC count (109/l) | 15.0 (13.1) | 13.1 (15.9) | -1.9 (p=0.26) |
|  | ^†^Platelet count (105/µl) | 2.0 (1.3) | 1.7 (1.9) | -0.3 (p=0.69) |
|  | ^†^C-reactive protein (mg/l) | 4.9 (24.7) | 6.7 (26.3) | +1.8 (p=0.60) |
|  | ^†^Urea (mg/dl) | 24.0 (17.0) | 18.0 (14.0) | -6.0 (p=0.44) |
|  | Creatinine (mg/dl) | 0.7 (0.1) | 0.7 (0.1) | 0.0 (-0.1,0.0) |
|  | Sodium (mEq/l) | 137.4 (5.8) | 131.9 (6.0) | -5.5 (-9.1,-1.8)* |
|  | Potassium (mEq/l) | 5.0 (0.7) | 4.9 (0.8) | -0.1 (-0.6,0.4) |
|  | ^†^SGPT (U/l) | 20.0 (21.0) | 34.5 (57.0) | +14.5 (p=0.19) |
|  | ^†^PT (seconds) | 15.0 (4.0) | 15.0 (4.0) | +0.0 (p=0.86) |
|  | APTT (seconds) | 40.0 (8.3) | 42.0 (11.5) | +2.0 (-4.2,8.1) |
