# Supplementary material for: Neonatal Encephalopathic Cerebral Injury in South India Assessed by Perinatal Magnetic Resonance Biomarkers and Early Childhood Neurodevelopmental Outcome
Source: PLoS One. 2014 Feb 5;9(2):e87874. doi: 10.1371/journal.pone.0087874 (PMC3914890; doi:10.1371/journal.pone.0087874)
Supplement: Table S3 — Clinical features and brain injury of surviving infants who attended 3½ year follow-up or were lost to follow-up. Values are proportion (%) unless otherwise indicated. CI = confidence interval; WM = white matter; BGT = basal ganglia and thalami; PLIC = posterior limb of the internal capsule. (DOCX) [file pone.0087874.s009.docx]

Table S3.

| Characteristic | Attended follow-up (n=38) | Lost to follow-up (n=10) | Difference (95% CI) |
| --- | --- | --- | --- |
| No encephalopathy (d3 Sarnat) | 2/38 (5%) | 1/10 (10%) | +5% (-10%,35%) |
| Mild encephalopathy (d3 Sarnat) | 24/38 (63%) | 6/10 (60%) | -3% (-35%,25%) |
| Moderate encephalopathy (d3 Sarnat) | 10/38 (26%) | 3/10 (30%) | +4% (-21%,36%) |
| Severe encephalopathy (d3 Sarnat) | 2/38 (5%) | 0/10 (0%) | -5% (-17%,23%) |
| Abnormal neurological exam at discharge | 16/38 (42%) | 5/10 (50%) | +8% (-23%,38%) |
| Moderate/severe cortical injury | 9/35 (26%) | 1/9 (11%) | -15% (-33%,20%) |
| Moderate/severe WM injury | 19/35 (54%) | 3/9 (33%) | -21% (-47%,14%) |
| Moderate/severe BGT injury | 7/35 (20%) | 1/9 (11%) | -9% (-27%,25%) |
| Moderate/severe PLIC injury | 4/35 (11%) | 1/9 (11%) | 0% (-18%,33%) |
