# Supplementary material for: Neonatal Encephalopathic Cerebral Injury in South India Assessed by Perinatal Magnetic Resonance Biomarkers and Early Childhood Neurodevelopmental Outcome
Source: PLoS One. 2014 Feb 5;9(2):e87874. doi: 10.1371/journal.pone.0087874 (PMC3914890; doi:10.1371/journal.pone.0087874)
Supplement: Table S4 — Characteristics and outcomes of neonatal encephalopathy for infants undergoing whole-body therapeutic hypothermia or normothermia. Values are mean (standard deviation) or proportion (%). †Sepsis = Clinical sepsis with elevated C-reactive protein with or without positive blood culture within three days of birth. ‡Abnormal outcome = cerebral palsy; visual or hearing impairment; evidence of seizures or use of anti-epileptic medication at age 3½ years; slowed head growth; or a composite motor score <82 or composite cognitive score <85 on Bayley III. (DOCX) [file pone.0087874.s010.docx]

Table S4.

| Characteristic | Therapeutic hypothermia (n=17) | Normothermia (n=37) | Difference (95% CI) |
| --- | --- | --- | --- |
| Gestational age (weeks+days) | 38+0 (1+2) | 38+3 (1+1) | +3 (-2,8) |
| Birth weight (g) | 2978 (402) | 2856 (395) | -122 (-355,112) |
| Early onset sepsis^†^ | 9/17 (53%) | 9/37 (24%) | -29% (-52%,-2%) |
| Moderate/severe WM injury | 8/13 (62%) | 14/31 (45%) | -16% (-43%,15%) |
| Moderate/severe BGT injury | 5/13 (39%) | 3/31 (10%) | -29% (-56%,-3%) |
| Moderate/severe cortical injury | 6/13 (46%) | 4/31 (13%) | -33% (-59%,-5%) |
| PLIC injury | 3/13 (23%) | 2/31 (7%) | -17% (-44%,4%) |
| Died | 4/17 (24%) | 2/37 (5%) | -18% (-42%,0%) |
| Abnormal 3½ year outcome^‡^ | 4/12 (33%) | 12/26 (46%) | +13% (-20%,40%) |
