# Supplementary material for: Neonatal Encephalopathic Cerebral Injury in South India Assessed by Perinatal Magnetic Resonance Biomarkers and Early Childhood Neurodevelopmental Outcome
Source: PLoS One. 2014 Feb 5;9(2):e87874. doi: 10.1371/journal.pone.0087874 (PMC3914890; doi:10.1371/journal.pone.0087874)
Supplement: Table S5 — Sensitivity and specificity of conventional MR imaging in identifying infants with cerebral palsy or low Bayley III scores (<82 for composite motor, <85 for composite cognitive) at 3½ years. (DOCX) [file pone.0087874.s011.docx]

Table S5.

| Diagnostic criterion | Sensitivity (95% CI) | Specificity (95% CI) |
| --- | --- | --- |
| Low PLIC T1 MR imaging signal intensity | 0.43 (0.16,0.75) | 0.96 (0.82,0.99) |
| Moderate/Severe BGT injury | 0.43 (0.16,0.75) | 0.86 (0.69,0.94) |
| Severe WM injury | 0.43 (0.16,0.75) | 0.89 (0.73,0.96) |
| Any of the above | 0.57 (0.25,0.84) | 0.79 (0.61,0.90) |
